# *LMNA*-related muscular dystrophy: Identification of variants in alternative genes and personalized clinical translation

**DOI:** 10.3389/fgene.2023.1135438

**Published:** 2023-03-24

**Authors:** Sergi Cesar, Monica Coll, Victoria Fiol, Anna Fernandez-Falgueras, Jose Cruzalegui, Anna Iglesias, Isaac Moll, Alexandra Perez-Serra, Estefanía Martínez-Barrios, Carles Ferrer-Costa, Bernat del Olmo, Marta Puigmulè, Mireia Alcalde, Laura Lopez, Ferran Pico, Rubén Berrueco, Josep Brugada, Irene Zschaeck, Daniel Natera-de Benito, Laura Carrera-García, Jessica Exposito-Escudero, Carlos Ortez, Andrés Nascimento, Ramon Brugada, Georgia Sarquella-Brugada, Oscar Campuzano

**Affiliations:** ^1^ Pediatric Arrhythmias, Inherited Cardiac Diseases and Sudden Death Unit, Hospital Sant Joan de Déu, Barcelona, Spain; ^2^ Arrítmies pediàtriques, Cardiologia Genètica i Mort sobtada, Malalties Cardiovasculars en el Desenvolupament, Institut de Recerca Sant Joan de Déu, Esplugues de Llobregat, Barcelona, Spain; ^3^ European Reference Network for Rare, Low Prevalence and Complex Diseases of the Heart (ERN GUARD-Heart), Amsterdam, Netherlands; ^4^ Cardiovascular Genetics Center, University of Girona-IDIBGI, Girona, Spain; ^5^ Centro de Investigación Biomédica en Red, Enfermedades Cardiovasculares (CIBERCV), Madrid, Spain; ^6^ Medical Science Department, School of Medicine, Universitat de Girona, Girona, Catalonia, Spain; ^7^ Pediatric Hematology Service, Hospital Sant Joan de Déu Barcelona, Institut de Recerca Pediàtrica, Hospital Sant Joan de Déu de Barcelona (IRP-HSJD), Universitat de Barcelona, Barcelona, Spain; ^8^ Arrhythmia Section, Cardiology Service, Hospital Clínic, Barcelona, Spain; ^9^ Neuromuscular Unit, Department of Neurology, Hospital Sant Joan de Déu, Barcelona, Spain; ^10^ Investigación Aplicada en Enfermedades Neuromusculares Neurociències Institut de Recerca Sant Joan de Déu, Esplugues de Llobregat, Spain; ^11^ Instituto Nacional de Investigación Biomédica de Enfermedades Raras (CIBERER), Instituto de Salud Carlos III, Madrid, Spain; ^12^ Cardiology Department, Hospital Josep Trueta, Girona, Catalonia, Spain

**Keywords:** sudden cardiac death, laminopathies, muscular dystrophy, genetics, genetic diagnostic

## Abstract

**Background:** Laminopathies are caused by rare alterations in *LMNA*, leading to a wide clinical spectrum. Though muscular dystrophy begins at early ages, disease progression is different in each patient. We investigated variability in laminopathy phenotypes by performing a targeted genetic analysis of patients diagnosed with *LMNA*-related muscular dystrophy to identify rare variants in alternative genes, thereby explaining phenotypic differences.

**Methods:** We analyzed 105 genes associated with muscular diseases by targeted sequencing in 26 pediatric patients of different countries, diagnosed with any *LMNA*-related muscular dystrophy. Family members were also clinically assessed and genetically analyzed.

**Results:** All patients carried a pathogenic rare variant in *LMNA*. Clinical diagnoses included Emery-Dreifuss muscular dystrophy (EDMD, 13 patients), *LMNA*-related congenital muscular dystrophy (L-CMD, 11 patients), and limb-girdle muscular dystrophy 1B (LGMD1B, 2 patients). In 9 patients, 10 additional rare genetic variants were identified in 8 genes other than *LMNA*. Genotype-phenotype correlation showed additional deleterious rare variants in five of the nine patients (3 L-CMD and 2 EDMD) with severe phenotypes.

**Conclusion:** Analysis f known genes related to muscular diseases in close correlation with personalized clinical assessments may help identify additional rare variants of *LMNA* potentially associated with early onset or most severe disease progression.

## 1 Introduction

Muscular dystrophies caused by deleterious variants in the *LMNA* gene are very rare (<1 per 1,000,000; ORPHA:157973). Cervico-axial and scapuloperoneal weakness, joint contractures, and thoracic lordosis associated with a dystrophic muscle biopsy and variably elevated creatine kinase levels are usually associated with these severe entities (Quijano-Roy et al., 2008). Sudden death (SD) also is common in these patients, mainly due to malignant arrhythmias concomitant with heart alterations (Kumar et al., 2016).

Through alternative splicing, *LMNA* encodes proteins lamin A and C, intermediate filaments that are required during development and cell differentiation and are components of the nuclear envelope (Bonne et al., 2003; Paul and Fulka, 2022). More than 500 rare genetic alterations in *LMNA* have been found to be responsible for a group of diseases called laminopathies (Worman and Bonne, 2007; Dittmer and Misteli, 2011). The most common laminopathies are *LMNA*-related congenital muscular dystrophy (L-CMD) (OMIM: 613205), Emery–Dreifuss muscular dystrophy (EDMD) (OMIM: 181350), limb-girdle muscular dystrophy type 1B (LGMD-1B) (OMIM: 159001), and dilated cardiomyopathy with conduction defects (DCM-CD) (OMIM: 115200). A range of phenotypes have been reported in patients carrying deleterious rare *LMNA* variants (Bertrand et al., 2011; Worman, 2012; Carboni et al., 2013a; Carboni et al., 2013b; Ben Yaou et al., 2021). It has been suggested that different phenotypes can be explained by post-transcriptional modifications of the nuclear envelope/lamina proteins (Maraldi et al., 2011; Zheng et al., 2022). Although *LMNA* is accepted as the main cause of disease, the observed phenotype differences remain poorly understood, mainly at early stages of the disease. Genetic background is suggested to be responsible for these phenotypic differences in disease onset and progression, although no studies investigating the role of additional genetic variants have been reported so far. Understanding genetic background variability can facilitate the early diagnosis of *LMNA*-related muscular dystrophy, which is important for prevention of SD, rehabilitation management, and genetic counseling (Charniot et al., 2003; Choi et al., 2019; Murofushi et al., 2022).

In the present study, we performed a targeted genetic analysis and personalized genotype-phenotype interpretation in families diagnosed with *LMNA*-related muscular dystrophies. We identified rare alterations in genes other than *LMNA* that may help explain the differences in disease onset and phenotype progression.

## 2 Materials and methods

### 2.1 Cohort

The study was approved by the Ethics Committee of the Hospital Josep Trueta (Girona, Spain) and Hospital Sant Joan de Déu (Barcelona, Spain), following the Helsinki II declaration. Written informed consent to participate in this study was provided by the participants’ legal guardians. Written informed consent was also obtained from all relatives included in the study.

The study enrolled 26 pediatric patients previously diagnosed with any type of *LMNA* muscular dystrophy (2014–2020) and carrying a definite pathogenic rare variant in *LMNA*. We retrospectively collected all available data from each patient’s first clinical contact up to their enrollment in our study. Clinical evaluation of index cases included a complete physical examination by a pediatric neurologist, neuromuscular specialist, and pediatric cardiologist. Non-pediatric relatives enrolled in our study also were clinically assessed. Saliva or peripheral blood samples were obtained from each patient as well as all available family members. All individuals were clinically assessed at Hospital Sant Joan de Déu (Barcelona, Catalonia, Spain). The complete pedigree of each family was obtained, including history of neuromuscular and cardiac diseases, syncope, and unexplained deaths.

### 2.2 Genetic analysis

Genomic DNA was analyzed using next-generation sequencing (NGS). A total of 105 genes involved in neuromuscular diseases and risk of malignant cardiac arrhythmias were analyzed (*ACTA1, AGRN, ANO5, B3GALNT2, B4GAT1, BAG3, BIN1, CAPN3, CAV3, CCND3, CFL2, CHAT, CHKB, CHRNA1, CHRNB1, CHRND, CHRNE, CNTN1, COL6A1, COL6A2, COL6A3, COLQ, DAG1, DES, DMD, DNAJB6, DNM2, DOK7, DPAGT1, DPM1, DPM2, DPM3, DSC2, DSG2, DSP, DYSF, EMD, FHL1, FKRP, FKTN, FLNC, FOS, FXN, GAA, GFPT1, GMPPB, GOSR2, HRAS, ISPD, ITGA7, KBTBD13, KCNQ1, KCNH2, LAMA2, LAMP2, LARGE, LDB3, LMNA, MOK2, MTM1, MUSK, MYF6, MYOT, MYH7, MYBPC3, NEB, NESPRIN2, NUP88, PCNA, PLEC, PKCA, PKP2, POMGNT1, POMGNT2, POMK, POMT1, POMT2, RAPSN, RYR1, RYR2, SCN4A, SCN5A, SEPN1, SGCA, SGCB, SGCD, SGCG, SLC25A4, SREBP, TAZ, TCAP, TMEM5, TMEM43, TMPO, TNNT1, TNNI3, TNNT2, TNPO3, TPM2, TPM3, TRIM32,* and *TTN*). All gene isoforms described in Ensembl 75 (www.ensembl.org) that have been linked with either a RefSeq code (www.ncbi.nlm.nih.gov/refseq) or CCDS (www.ncbi.nlm.nih.gov/CCDS) were included. Sequence data coordinates were based on UCSC human genome version hg19 (NCBI GRCh37 built). Biotinylated cRNA probe solution was used as a capture probe (Agilent Technologies, Santa Clara, CA, United States). Probes were designed using eArray (Agilent Technologies).

Non-common genetic variants [minor allele frequency (MAF) < 1%] identified throughout NGS analysis were confirmed using Sanger sequencing. Exons and exon–intron boundaries of each gene were amplified (Verities PCR, Applied Biosystems, Austin, TX, United States), and the resulting PCR products were purified (Exosap-IT, Affymetrix Inc., USB Products, Cleveland, OH, United States) and directly sequenced in both directions (Big Dye Terminator v3.1 and 3130XL Genetic Analyzer, both from Applied Biosystems). The Posterior SeqScape Software v2.5 (Life Technologies, Carlsbad, CA, United States) was used to compare results with the reference sequence from hg19. The identified rare variants were contrasted with the Human Gene Mutation Database (www.hgmd.cf.ac.uk/ac/index.php) and Genome Aggregation Database (gnomAD) (www.gnomad.broadinstitute.org). To detect copy number variation (CNV), we looked for significant differences between expected and obtained normalized coverage for a given sample in a region of interest. Several samples were analyzed to corroborate similar levels of coverage between samples. All CNVs were compared with the CNV Control database (www.gwas.biosciencedbc.jp/cgi-bin/cnvdb/cnv_top.cgi), Database of Genomic Variants (www.dgv.tcag.ca/dgv/app/home), DECIPHER (www.decipher.sanger.ac.uk), and gnomAD (www.gnomad.broadinstitute.org). Rare variants that were potentially deleterious and confirmed in the index case were analyzed using the Sanger method in the relatives.

Each rare variant was classified following current recommendations of the American College of Medical Genetics and Genomics (ACMG) (Richards et al., 2015). A vast majority of pathogenic (P) variants are extremely rare (<0.01%). All available data concerning each rare genetic variant was updated until submission time (June, 2022). Variants classified as Variant of Unknown Significance (VUS) in alternative genes were further sub-classified. Variants identified showed no reported MAF or low MAF. Certain association with any neuromuscular disease were considered as VUS with highly suspicious Likely Pathogenic role (VUS-LP); thus, they were included to clarify their potential role in clinical practice. To avoid bias, five investigators independently investigated genetic data concerning each analyzed variant in our study. Finally, all investigators discussed data included in each item of the ACMG and consensus as well as final classification of all rare variants.

## 3 Results

### 3.1 Cohort

**FIGURE 1:**
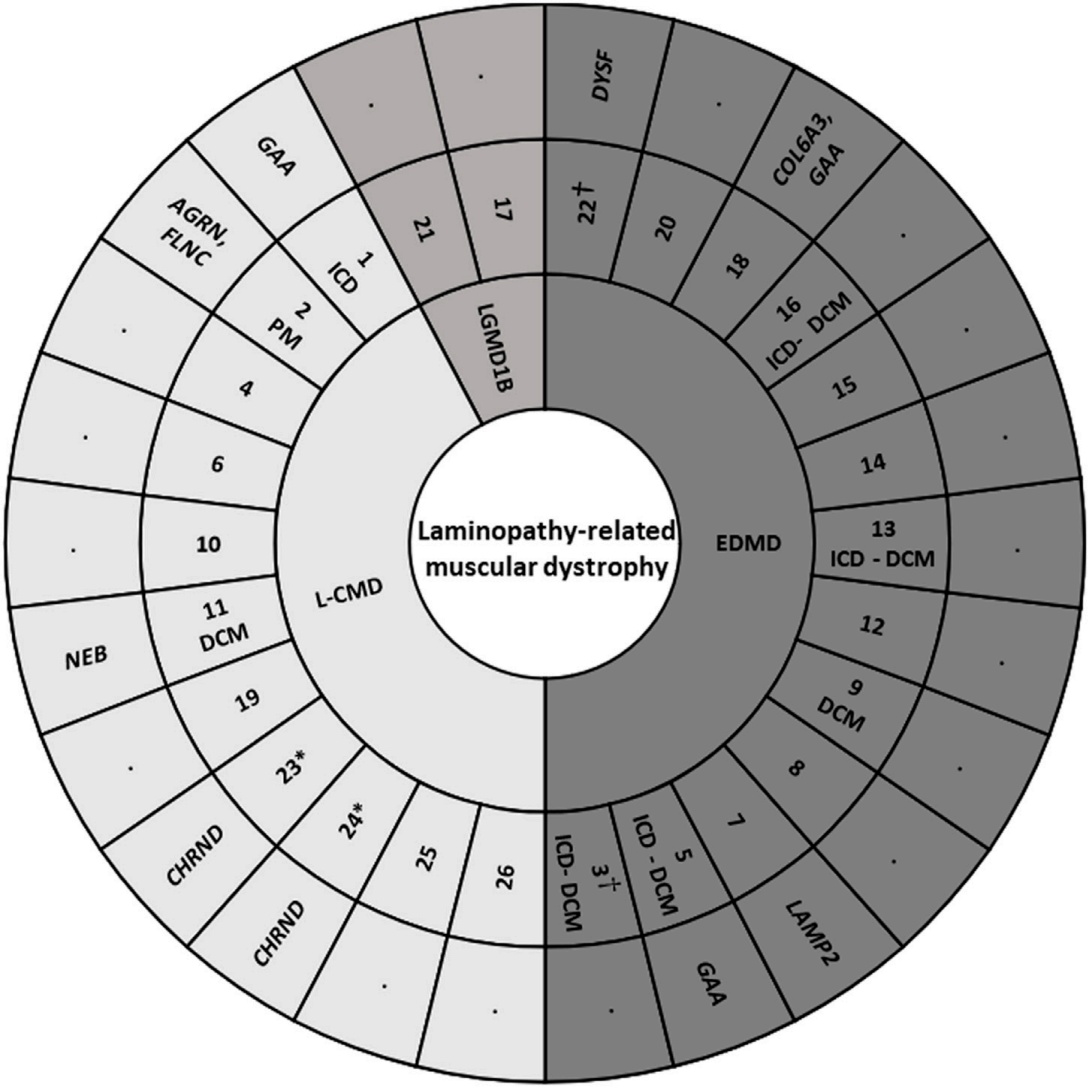
Cohort of index cases. DCM, dilated cardiomyopathy. EDMD, Emery-Dreifuss muscular dystrophy. ICD, implantable cardioverter defibrillator. L-CMD, *LMNA*-related congenital muscular dystrophy. LGMD1B, Limb-girdle muscular dystrophy 1B. PM, pacemaker. *, twins. †, sudden death.

Our study included 26 pediatric patients (mean age 8.2 years at enrollment; IQR, 4–12.5 years; 53.8% males) of 25 families, with a total of 76 individuals (26 index cases and 50 relatives). Two of the index cases enrolled were female monozygotic twins (index cases 23 and 24). Families were originally from Spain (12), United Kingdom (3), United States (3), Australia (2), Canada (1), France (1), Greece (1), Russia (1), and Argentina (1). No consanguinity occurred in any of families. No potential common ancestor was identified after the family interviews. All index cases accomplished with clinical criteria for LMNA-related muscular disease: 11 Patients (42.3%) were L-CMD, 13 (50%) were EDMD, and 2 (7.7%) presented as LGMD1B. Early-onset skeletal muscle impairment before 2 years of age was detected in 23 of the 26 cases (88.4%) (Figure 1). Clinical assessment was performed in all patients included in our cohort, confirming previous diagnosis for each *LMNA*-related muscular disease.

### 3.2 Genetic analysis

Our NGS analysis showed an average call rate of 99.25% achieved at 30x coverage. The median coverage per sample was 892 (749–1286). An average of four failed whole exons occurred in each sample, and all these exons were amplified using Sanger sequencing. All rare variants (MAF < 1%) were confirmed also using Sanger sequencing, discarding false positive signal. No CNV were identified in any of genes analyzed, including *LMNA.* Previous karyotype (performed at time of diagnosis, out of our centre) also discarded any large chromosomic alteration in all patients included in our cohort.

### 3.3 Rare variants in LMNA

**FIGURE 2:**
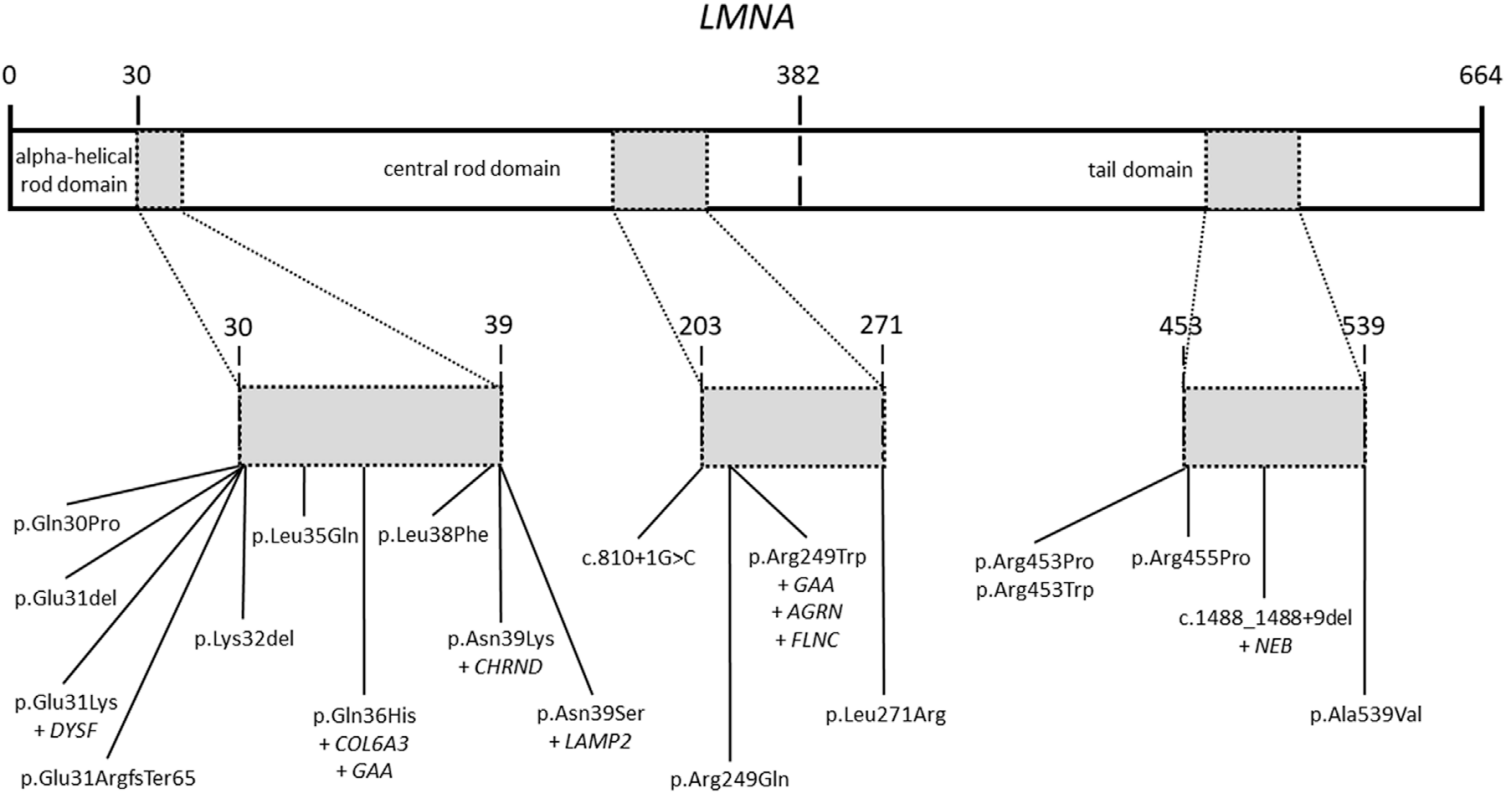
Rare variants in the *LMNA* gene. Domains of the *LMNA* gene including rare variants identified in our patients. All rare variants are located in three main zones of the gene.

All patients included in our study had a previous genetic analysis of the *LMNA* gene. This previous analysis identified rare variants in this gene as potential cause of the disease. Our targeted-gene panel analysis confirmed all previous rare variants in the *LMNA* gene (Figure 2). No additional rare or common variant classified as pathogenic (P) or likely pathogenic (LP) were identified in *LMNA*. As above mentioned, previous genetic analysis identified rare variants in the *LMNA* gene, which were classified according to available data at the moment of genetic analysis was performed. Rare variants in *LMNA* were reclassified following ACMG guidelines and accordingly to current data available (June, 2022). All *LMNA* variants remain classified as P or LP, without any modification in comparison to previous genetic report.

A total of 19 *LMNA* rare variants were identified (17 exonic and 2 intronic). Of exonic rare variants, 4 were delins and 13 missense. All variants were identified in heterozygous state (Figure 2). Eleven rare variants (57.89%) were classified as LP and 7 (36.84%) as definitively P. The most frequent rare variant identified in *LMNA* was p.Arg249Trp (5 patients, 19.23%), as previously reported (Quijano-Roy et al., 2008; Pasqualin et al., 2014; Heller et al., 2017; Ben Yaou et al., 2021; Fan et al., 2021; Jedrzejowska et al., 2021). These 5 patients were diagnosed with L-CMD (4 patients -index case 1, 2, 6, and 26-) and EDMD (1 patient -index case 14-). Other two rare variants were identified two times in different patients each one (p.Asn39Ser, patients 3 and 7; p.Arg453Trp, patients 20 and 21). Both rare variants were also previously reported (Pasqualin et al., 2014; Ben Yaou et al., 2021; Bennett et al., 2021; Fan et al., 2021; Jedrzejowska et al., 2021). A total of 6 cases showed DCM (patient 3, 5, 9, 11, 13, and 16), five of them diagnosed with EDMD and only one with L-CMD (patient 11). Among the patients with ICD (patients 1, 3, 5, 13, and 16), four were diagnosed with EDMD and one with L-CMD (patient 1). Index case number 2, diagnosed with L-CMD and carrying a pacemaker (PM), showed prolonged asystole episodes. Unfortunately, two cases died after inclusion in our study (patient 3 due to rapidly progressive heart failure despite optimal treatment and ICD carrier and 22due to severe respiratory infection) (Tables 1–3) (Figure 1). Finally, focused on intronic *LMNA* rare variant (c.810 + 1G>C) in patient 17, the clinical diagnostic was LGMD1B. The patient was not a carrier of any other rare variant in either the *LMNA* gene or any other gene. In addition, none of relatives showed any clinical symptom (Table 1).

**TABLE 1:** *LMNA*-related muscular dystrophy patients, major cardiac end points and rare *LMNA* variants.

Patient	Sex	Age diagnosis	Phenotype	DCM	Device	*LMNA* variants (LP, P)	Other variants
1	M	11	L-CMD	No	ICD	c.745C > T (p.Arg249Trp)	*GAA*
2	M	2	L-CMD	No	PM	c.745C > T (p.Arg249Trp)	*AGRN, FLNC*
3†	F	16	EDMD	Yes	ICD	c.116A > G (p.Asn39Ser)	None
4	F	9	L-CMD	No	No	c.91_93delGAG (p.Glu31del)	None
5	M	11	EDMD	Yes	ICD	c.1358G > C (p.Arg453Pro)	*GAA*
6	M	9	L-CMD	No	No	c.745C > T (p.Arg249Trp)	None
7	M	3	EDMD	No	No	c.116A > G (p.Asn39Ser)	*LAMP2*
8	F	8	EDMD	No	No	c.746G > A (p.Arg249Gln)	None
9	M	7	EDMD	Yes	No	c.91delG (p.Glu31ArgfsTer65)	None
10	F	2	L-CMD	No	No	c.89A > C (p.Gln30Pro)	None
11	F	3	L-CMD	Yes	No	c.1488_1488+9del	*NEB*
12	M	15	EDMD	No	No	c.1616C > T (p.Ala539Val)	None
13	M	15	EDMD	Yes	ICD	c.112C > T (p.Leu38Phe)	None
14	F	8	EDMD	No	No	c.745C > T (p.Arg249Trp)	None
15	M	13	EDMD	No	No	c.812T > G (p.Leu271Arg)	None
16	M	15	EDMD	Yes	ICD	c.1364G > C (p.Arg455Pro)	None
17	F	11	LGMD1B	No	No	c.810 + 1G > C	None
18	F	3	EDMD	No	No	c.108G > T (p.Gln36His)	*COL6A3, GAA*
19	M	3	L-CMD	No	No	c.104T > A (p.Leu35Gln)	None
20	F	17	EDMD	No	No	c.1357C > T (p.Arg453Trp)	None
21	M	5	LGMD1B	No	No	c.1357C > T (p.Arg453Trp)	None
22†	F	18	EDMD	No	No	c.91G > A (p.Glu31Lys)	*DYSF*
23*,24*	F	5	L-CMD	No	No	c.117T > G (p.Asn39Lys)	*CHRND*
25	M	4	L-CMD	No	No	c.94_96delAAG (p.Lys32del)	None
26	M	4	L-CMD	No	No	c.745C > T (p.Arg249Trp)	None

DCM, dilated cardiomyopathy. EDMD, Emery-Dreifuss muscular dystrophy. F, Female. ICD, implantable cardioverter defibrillator. L-CMD, *LMNA*-related congenital muscular dystrophy. LGMD1B, Limb-girdle muscular dystrophy 1B. LP, Likely Pathogenic. M, Male. P, Pathogenic. PM, pacemaker. *, twins. †, sudden death.

**FIGURE 3:**
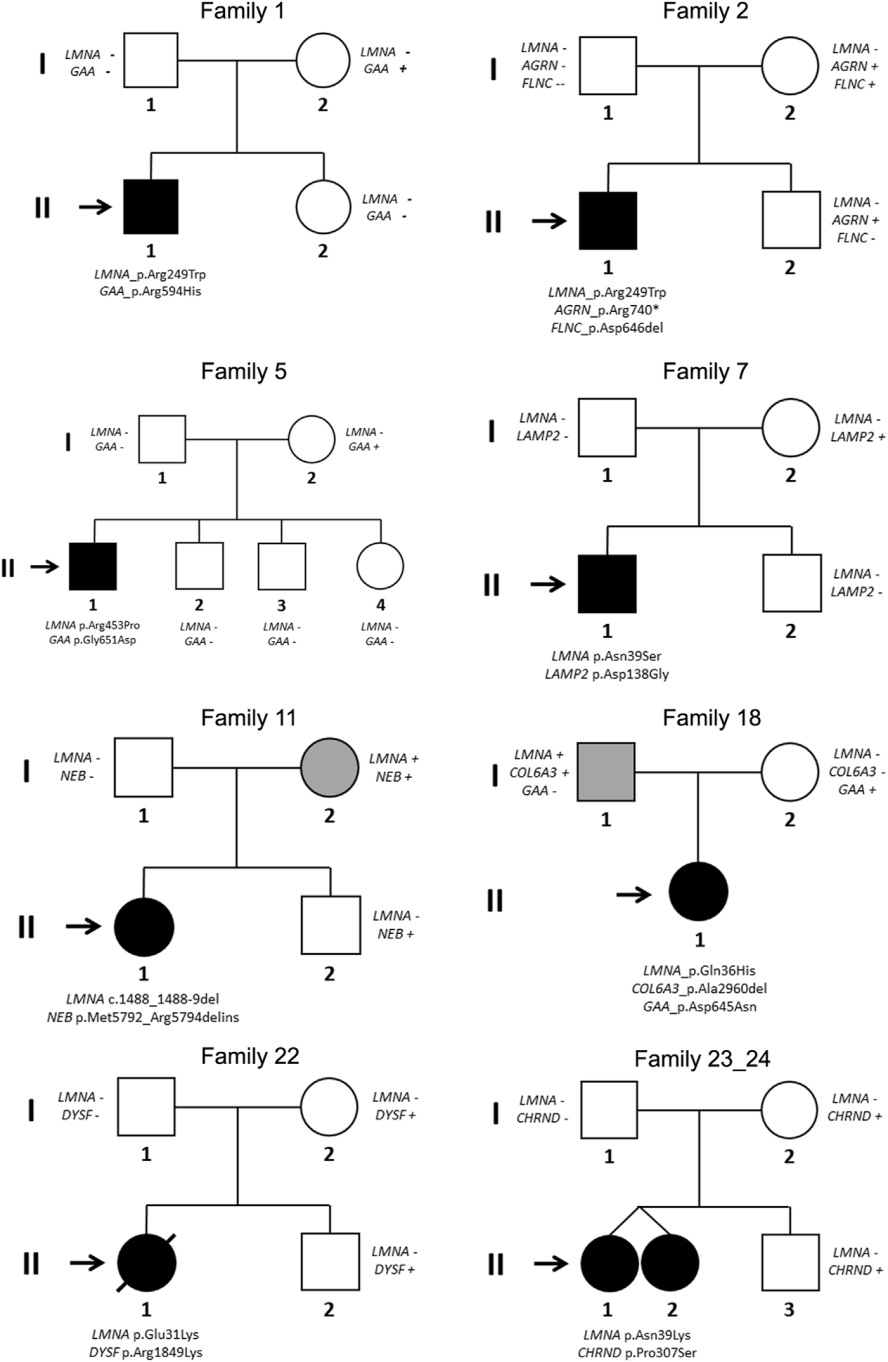
Pedigrees of families 1, 2, 5, 7, 11, 18, 22, and 23/24 (twins). Generations are indicated in the left side. Each individual of direct family linage is identified with a number. Clinically affected patients are shows in black, clinically unaffected patients are show in white, and mild phenotype is indicated in grey color. Slash indicates deceased. Index case is indicated with an arrow. Sign plus indicates carrier of the genetic variant. Minus sign indicates not carrier of the genetic variant.

Segregation of genetic variants in families showed that in 24 cases (92.3%), the rare variant in *LMNA* was *de novo* (including intronic variant in case number 17), as widely published (Ben Yaou et al., 2021; Jedrzejowska et al., 2021). Only in two families (index cases 11 and 18), one of parents carried the same deleterious variant in *LMNA*. In both these cases, the parents showed minor muscular impairment (Figure 3).

### 3.4 Additional rare variants

Eleven rare variants were identified in 8 genes (*AGRN, CHRND, COL6A3, DYSF, FLNC, GAA, LAMP2*, and *NEB*) encoding structural proteins. The *AGRN* gene encodes the protein Agrin, associated with Congenital myasthenic syndrome following an autosomal recessive (AR) pattern of inheritance. The *CHRND* gene encodes the Cholinergic receptor nicotinic delta subunit, mainly related to Myasthenic syndrome following autosomal dominant (AD) or AR pattern of inheritance. The *COL6A3* encodes Collagen type VI alpha 3 chain protein and deleterious variants are mainly associated with muscular dystrophy following both AD and AR patterns of inheritance. The *DYSF* gene encodes the protein Dysferlin, mainly associated with muscular dystrophy following an AR pattern of inheritance. The *FLNC* gene encodes the protein Filamin C, mainly associated wit cardiomyopathies following an AD pattern of inheritance. The *GAA* gene encodes Alpha Glucosidase and deleterious variants in this gene are related to Pompe disease following an AR pattern of inheritance. The *LAMP2*

Gene encodes the Lysosomal associated membrane protein 2, related to Danon disease following a X-linked pattern of inheritance. Finally, the *NEB* gene encodes the protein Nebulin, and pathogenic variants in this gene cause mainly myopathy following an AR pattern of inheritance (Table 2). At this point, it is important to remark that none of these proteins have a close relation to the *LMNA* gene according to consulted protein databases.

**TABLE 2:** Additional genes identified. AD, Autosomic Dominant; AR, Autosomic Recessive; XLD, X-Linked Dominant.

Genes	Location	Protein	Gene ID/HGNC/MIM	Disease/MIM/Inheritance
*GAA*	17q25.3	Alpha Glucosidase	2548/4065/606800	Pompe/232300/AR
*AGRN*	1p36.33	Agrin	375790/329/103320	Congenital myasthenic syndrome-8/615120/AR
*FLNC*	7q32.1	Filamin C	2318/3756/102565	Cardiomyopathy, familial hypertrophic/617047/AD
				Cardiomyopathy, familial restrictive/617047/AD
				Myopathy, distal/614065/AD
				Myopathy, myofibrillar/609524/AD
*LAMP2*	Xq24	Lysosomal associated membrane protein 2	3920/6501/309060	Danon/300257/XLD
*COL6A3*	2q37.3	Collagen type VI alpha 3 chain	1293/2213/120250	Bethlem myopathy/158810/AD, AR
				Dystonia/616411/AR
				Ullrich congenital muscular dystrophy/254090/AD, AR
*NEB*	2q23.3	Nebulin	4703/7720/161650	Arthrogryposis multiplex congenita/619334/AR
				Nemaline myopathy/256030/AR
*DYSF*	2p13.2	Dysferlin	8291/3097/603009	Miyoshi muscular dystrophy/254130/AR
				Muscular dystrophy, limb-girdle/253601/AR
				Myopathy, distal, with anterior tibial onset/606768/AR
*CHRND*	2q37.1	Cholinergic receptor nicotinic delta subunit	1144/1965/100720	Myasthenic syndrome, congenital, slow-channel/616321/AD
				Myasthenic syndrome, congenital, associated with acetylcholine receptor deficiency/616323/AR
				Multiple pterygium syndrome, lethal type/253290/AR
				Myasthenic syndrome, congenital, fast-channel/616322/AR

These rare variants were identified in 8 families −1, 2, 5, 7, 11, 18, 22, and 23/24- (30.76%). No other rare or common alterations (single variants, delins, or CNV), classified as P, LP, or VUS-LP, were identified in any of additional genes analyzed. All the new 11 rare variants were exonic and in heterozygous state. One variant was non-sense (patient 2), 3 not-in-frame deletions (patients 2, 11, and 18), 2 not-in-frame delins (patients 23/24 -twins-), and 5 missense (patients 1, 5, 7, 18, and 22). All rare variants were classified as LP or VUS-LP following ACMG recommendations. None rare variant was repeated in more than one patient except in twins (patients 23/24) (Tables 1–3) (Figure 1).

Segregation of genetic variants in families showed that none of these rare variants was *de novo*. Curiously, only two variants in *GAA* (p.Arg594His, CM121193-patient 1-and p.Asp645Asn, CM980804-patient 18-) were previously identified and associated with Glycogen Storage Disease (GSD) (Huie et al., 1998; McCready et al., 2007; Liu et al., 2014). None of parents (except families 11 and 18, due to *LMNA*) were previously diagnosed or showed any symptom associated with the genes identified in this study (Figure 3).

### 3.5 Genotype-phenotype correlation

Previous clinical diagnosis and follow-up showed 9 patients with more severe phenotypes (6 EDMD -patients 3, 5, 9, 13, 16 and 22- and 3 L-CMD -patients 1, 2 and 11-). All nine patients carried a rare missense deleterious variant in *LMNA* except two (patient 9-EDMD-and 11-L-CMD-) whose carried deletions (p.Glu31ArgfsTer65 and c.1488_1488+9del, respectively) (Tables 1–3).

**TABLE 3:** *LMNA* variants and related features found in our pediatric cohort.

Patient	Gene	Nucleotide change	Protein change	dbSNP	gnomAD (MAF%)	HGMD disease	ClinVar disease	ACMG score	Father	Mother	Other relatives
1	*GAA*	c.1781G>A	p.Arg594His	rs775450536	2/248732 (0.0008%)	CM121193, GSD	P, GSD	LP	-	+	Sister -
	*LMNA*	c.745C>T	p.Arg249Trp	rs121912496	NA	CM083718, MD	P/LP, MD	LP	-	-	Sister-
2	*AGRN*	c.2218C>T	p.Arg740Ter	NA	NA	NA	NA	LP	-	+	Sister +
	*FLNC*	c.1932delT	p.Cys644TrpfsTer27	NA	NA	NA	NA	LP	-	+	Sister-
	*LMNA*	c.745C>T	p.Arg249Trp	rs121912496	NA	CM083718, MD	P/LP, MD	LP	-	-	Sister-
3†	*LMNA*	c.116A>G	p.Asn39Ser	rs57983345	NA	CM083713, MD	P, CMT	P	-	-	NA
4	*LMNA*	c.91_93delGAG	p.Glu31del	rs864309525	NA	CD156162, MD	LP, MD	LP	-	-	NA
5	*GAA*	c.1952G > A	p.Gly651Asp	rs939350425	NA	NA	VUS	LP	-	+	Siblings -
	*LMNA*	c.1358G > C	p.Arg453Pro	rs267607598	NA	CM083716, MD	NA	LP	-	-	Siblings -
6	*LMNA*	c.745C > T	p.Arg249Trp	rs121912496	NA	CM083718, MD	P/LP, MD	LP	-	-	NA
7	*LAMP2*	c.413A > G	p.Asp138Gly	NA	NA	NA	NA	VUS-LP	-	+	Brother -
	*LMNA*	c.116A > G	p.Asn39Ser	rs57983345	NA	CM083713, MD	P, CMT	P	-	-	Brother -
8	*LMNA*	c.746G > A	p.Arg249Gln	rs59332535	NA	CM1617006, MD, ED	P, MD	P	-	-	NA
9	*LMNA*	c.91delG	p.Glu31ArgfsTer65	NA	NA	NA	NA	P	-	-	NA
10	*LMNA*	c.89A > C	p.Gln30Pro	NA	NA	NA	NA	LP	-	-	Brother -
11	*LMNA*	c.1487_1488 + 9del		NA	NA	CD1711480, MD	NA	P	-	+	Brother -
	*NEB*	c.17376_17381del	p.Met5792_Asp5794delinsIle	rs765184893	9/247156 (0.0036%)	NA	NA	VUS-LP	-	+	Brother +
12	*LMNA*	c.1616C > T	p.Ala539Val	NA	NA	NA	NA	LP	-	-	NA
13	*LMNA*	c.112C > T	p.Leu38Phe	NA	NA	NA	NA	LP	-	-	NA
14	*LMNA*	c.745C > T	p.Arg249Trp	rs121912496	NA	CM083718, MD	P/LP, MD	LP	-	-	NA
15	*LMNA*	c.812T > G	p.Leu271Arg	NA	NA	NA	NA	LP	-	-	NA
16	*LMNA*	c.1364G > C	p.Arg455Pro	rs267607597	NA	CM111722, MD	NA	LP	-	-	NA
17	*LMNA*	c.810 + 1G > C		rs267607632	NA	NA	P, MD	P	-	-	NA
18	*COL6A3*	c.8883delA	p.Lys2961AsnfsTer40	NA	NA	NA	NA	LP	+	-	NA
	*GAA*	c.1933G>A	p.Asp645Asn	rs368438393	2/242414 (0.0008%)	CM980804, GSD	LP, GSD	LP	-	+	NA
	*LMNA*	c.108G>T	p.Gln36His	NA	NA	NA	NA	LP	+	-	NA
19	*LMNA*	c.104T>A	p.Leu35Gln	NA	NA	NA	NA	LP	-	-	NA
20	*LMNA*	c.1357C>T	p.Arg453Trp	rs58932704	NA	CM990813, MD	P/LP, MD	LP	-	-	NA
21	*LMNA*	c.1357C>T	p.Arg453Trp	rs58932704	NA	CM990813, MD	P/LP, MD	LP	-	-	NA
22†	*DYSF*	c.5546G>A	p.Arg1849Lys	rs786205084	NA	NA	LP, MD	LP	-	+	Brother +
	*LMNA*	c.91G>A	p.Glu31Lys	rs1228406418	NA	CM123360, MD	P, CMT	LP	-	-	Brother -
23,24 twins	*CHRND*	c.919_920delCCinsAG	p.Pro307Ser	NA	NA	NA	NA	VUS-LP	-	+	Brother +/Twin +
	*LMNA*	c.117T>G	p.Asn39Lys	NA	NA	CM156123, MD	NA	LP	-	-	Brother -/Twin +
25	*LMNA*	c.94_96delAAG	p.Lys32del	rs60872029	NA	CD033712, MD, ED	P, MD	P	-	-	NA
26	*LMNA*	c.745C>T	p.Arg249Trp	rs121912496	NA	CM083718, MD	P/LP, MD	LP	-	-	NA

dbSNP, single nucleotide polymorphism database. ACMG, score, the American College of Medical Genetics and Genomics score. ClinVar, Clinically relevant Variation database. CMT, Charcot-Marie-Tooth. ED, Emery-Dreifuss. GnomAD (MAF%), Genome Aggregation Database (minor allele frequency %). GSD, Glycogen Storage Disease. HGMD, the Human Gene Mutation Database. LP, Likely Pathogenic. MD, muscular dystrophy. NA, not available. P, Pathogenic. †, sudden death. VUS-LP, variant of unknown significance with highly suspicious likely pathogenic role.

Six patients diagnosed with EDMD (patients 3, 5, 9, 13, 16, and 22) showed different phenotypes: DCM (patient 9) or DCM and carried an ICD (patients 3, 5, 13, and 16). Curiously, patient 22 did not show any risk factor but died suddenly. Patient 3 also died suddenly despite carrying an ICD. Only patient 9 carried a rare variant in *LMNA* (c.91delG) while all other patients carried a missense variant in the same gene. Concerning additional rare variants in other genes, only patient 5 carried a rare missense variant (c.1952G>A) in *GAA*, inherited from their unaffected mother (Figure 1).

Three patients diagnosed with L-CMD (patients 1, 2, and 11) also showed different phenotypes: DCM (patient 11) or carried an ICD (patient 1) or a PM (patient 2) but without DCM. Curiously, patients 1 and 2 (no family relationship) carried the same missense rare variant in *LMNA* (p.Arg249Trp) and patient 11 carried an intronic rare variant (c.1488_1488+9del). Additionally, all patients carried at least one rare variant: patient 1 in *GAA* (p.Arg594His) inherited from their mother and without any symptom to date, patient 2 carried two rare variants, one in *AGRN* and other in *FLNC* (p.Arg740Ter and p.Cys644TrpfsTer27, respectively), both rare variants inherited from their healthy mother, and patient 11 carried a rare variant in *NEB* (p.Met5792_Arp5794delinsIle), inherited from their mother who showed a mild neuromuscular affectation but their brother carried the same variant in *NEB* without any symptom diagnosed to date (Tables 1–3) (Figures 1, 2).

## 4 Discussion

A cohort of 26 patients diagnosed with *LMNA*-related muscular diseases were analyzed for the increasing number of additional rare alterations in other genes which may be involved in phenotype differences. We identified that 56% of patients with most severe phenotypes, mainly diagnosed with L-CMD, carried a deleterious rare variant in the *LMNA* gene, but also an additional deleterious rare variant in another gene associated with NMD, and played a potential role in early onset and disease progression.

Only a few cohorts of cases diagnosed with *LMNA*-related muscular diseases have been published to date, all following an autosomal dominant pattern of inheritance, as occurs in our study. In 2007, a cohort including 27 patients (EDMD, 56%; CMD, 15%; LGMD, 30%) (Benedetti et al., 2007). Other cohort was published in 2014, and included 78 cases diagnosed with *LMNA*-related myopathies (EDMD, 21%; L-CMD, 33%; LGMD1B, 46%) (Maggi et al., 2014). In addition, a cohort of 84 patients diagnosed with *LMNA*-related muscular dystrophy were also analyzed (EDMD, 38%; L-CMD, 49%; LGMD1B, 13%) (Fan et al., 2021). Our study shows similar percentages of *LMNA*-related muscular diseases (EDMD, 50%; L-CMD, 42%; LGMD1B, 8%). Recently, the largest cohort including 151 L-CMD patients was also published (Ben Yaou et al., 2021), reinforcing the necessity of anticipatory care of respiratory and cardiac assessment due to rapid progression of symptoms especially in L-CMD. As these are ultra-rare diseases, it is difficult to obtain patients with a definite diagnosis. Various forms of skeletal muscle laminopathies may overlap with each other, creating a phenotypic continuum, as recently reported in a cohort of 15 children with initial symptoms visible during first year of life, included hypotonia, poor head control, or delayed motor development (Jedrzejowska et al., 2021). In addition, involving large number of cases of different ethnic origin, as done in our study for the first time, is crucial to clarify role of genetic background in these ultra-rare diseases in onset as well as progression of disease. Currently, it is widely accepted *LMNA*-related muscular diseases as monogenic entities due to a single P rare variant in the *LMNA* gene. However, due to reported differences in severity of phenotypes, existence of alternative rare variants as phenotype modifiers is suspected despite not reported to date. Our study aims to solve this gap in *LMNA*-related muscular diseases.

It is widely accepted that early diagnosis of *LMNA*-related muscular diseases is key for appropriate clinical management (Charniot et al., 2003), particularly in L-CMD (Ben Yaou et al., 2021). In addition, clinical familial history and close genotype-phenotype correlation can help clarify the role of genetic variants in onset as well as progression of disease (Cotta et al., 2019; Ben Yaou et al., 2021). Therefore, existence of additional rare genetic modifiers has been suggested as an explanation for clinical phenotype difference observed in families diagnosed with any type of laminopathy (Muntoni et al., 2006; Boudreau et al., 2012; Roncarati et al., 2013) despite no comprehensive genotype-phenotype study. Analysis of variant segregation in families can help unravel the role of the rare variants identified in this study. In summary, we report a targeted genetic analysis and segregation of variants in families, looking for additional rare variants in other genes than *LMNA*, which could explain the phenotypic differences in LMNA-related muscular diseases.

### 4.1 Genotype-phenotype correlation

All *LMNA* variants were deleterious, which was the main cause of the clinically diagnosed disease. However, different onset as well as disease progression seems to be modified by other variants, in concordance to previously suggested but not exhaustively analyzed to date. The variant p.Arg249Trp was identified in 5 patients (4 L-CMD and 1 EMD2). This variant was previously reported in several patients diagnosed with L-CMD (Ben Yaou et al., 2021). The variant was *de novo* in all reported cases and, in addition to early onset of muscular involvement, patients also showed cardiac involvement and malignant arrhythmias (Quijano-Roy et al., 2008; Komaki et al., 2011; Pasqualin et al., 2014; Ben Yaou et al., 2021; Fan et al., 2021; Jedrzejowska et al., 2021). In view of age-dependent penetrance for heart involvement due to deleterious variants in *LMNA*, a regular cardiological supervision should have been offered (Jedrzejowska et al., 2021), particularly in p.Arg249Trp carriers due to clinical severity (Ben Yaou et al., 2021).

Patients 1 and 2, diagnosed with L-CMD showed most severe phenotype and carried additional potentially deleterious rare variants (patient 1 in *GAA* and patient 2 in *AGRN* and *FLNC*). In patient 1, the *GAA*_p.Arg594His (CM121193) was previously reported and associated with GSD following an autosomal recessive pattern of inheritance (Liu et al., 2014). It was inherited from their asymptomatic mother and none showed any symptom of GSD or Pompe disease due to the heterozygous form. In patient 2, both variants were novel, inherited from their mother and classified as deleterious. The variants in *FLNC* are mainly associated with Hypertrophic cardiomyopathy (not observed in patient 2 or the mother) and variants in *AGRN* are mainly associated with congenital myasthenic syndrome following an autosomal recessive pattern of inheritance. Therefore, neither mother or sister showed any symptom of muscular weakness as both are carriers of the same heterozygotic variant. Therefore, the presence of any deleterious rare variant may explain the most severe muscular weakness and malignant arrhythmias observed in patients 1 and 2, in comparison to patients 6 and 26 (both showing same diagnosis and carrying the same *LMNA* variant but without aggressive phenotype). However, further molecular studies should be performed to unravel the pathophysiological mechanism involved in these potential phenotype modifications. Curiously, patient 14, diagnosed with EDMD and carrying the same variant p.Arg249Trp, showed a different phenotype possibly due to an unidentified alteration, reinforcing the targeted genetic analysis not only limited to the *LMNA* gene in patients diagnosed with *LMNA*-related muscular diseases. In addition, in p.Arg249, other deleterious variants were identified in patient 8, who was diagnosed with EDMD. This variant (p.Arg249Gln) was also previously reported in 3 infants showing slow muscular degeneration and slight arrhythmias (Bonne et al., 2000; Fan et al., 2021; Jedrzejowska et al., 2021), similar to this patient.

Patient 5, who showed a severe phenotype of EDMD, with DCM and implanted ICD due to malignant arrhythmias, carried two rare variants (*LMNA*_p.Arg453Pro and *GAA*_p.Gly651Asp). Both variants were novel. The *GAA* variant was inherited from the asymptomatic mother and no symptoms of GSD/Pompe disease in any carrier were observed due to widely-accepted recessive pattern of inheritance in this gene. Curiously, in the same aminoacid p.Arg453, another deleterious rare variant was previously reported as *de novo* in several cases (p.Arg453Trp) (Bonne et al., 2000; Fan et al., 2021; Jedrzejowska et al., 2021). In these reported cases, the diagnosis was EDMD concomitant with arrhythmias and slow muscular degeneration. Only in one case, the diagnosis was LGMD1B. In our cohort, two cases carried this rare variant *LMNA*_p.Arg453Trp, patient 20 diagnosed with EDMD and patient 21 diagnosed with LGMD1B (both showing slow muscular degeneration, without any arrhythmia or cardiac alteration).

Patient 7, diagnosed with EDMD but without any cardiac alteration, carried the deleterious *LMNA*_p.Asn39Ser variant. She carried an additional rare variant in the *LAMP2* gene, also identified in her mother. Rare variants in this gene are associated with Danon disease, following an X-linked pattern of inheritance. However, the mother did not show any symptom/phenotype related to Danon disease. The rare variant in *LMNA* was previously published in 5 patients, of which 3 were diagnosed with L-CMD and slow muscular weakness progression, and 2 with EDMD and no cardiac affectation (Pasqualin et al., 2014; Fan et al., 2021). In our cohort, patient 3 also carried the same variant but with a clinical diagnosis of EDMD. Despite no additional deleterious variant identified in any of all analyzed genes, DCM and malignant arrhythmias were documented. Unfortunately, this patient died due to rapidly progressive heart failure despite optimal treatment and being an ICD carrier. It suggests the targeted genetic analysis looking for other new genes not currently associated with any muscular diseases. Curiously, in the same aminoacid *LMNA*_p.Asn39, twins included in our cohort (patients 23/24) carried the deleterious variant (p.Asn39Lys) responsible of L-CMD diagnosed. This variant was recently reported in one case of L-CMD with slow muscular weakness progression and no cardiac affectation (Fan et al., 2021) and in two cases showing hypotonia, waddling gait and normal heart (Jedrzejowska et al., 2021), in concordance to our twins.

Patient 11, diagnosed with concomitant L-CMD and DCM, carried a *de novo* and novel deletion *LMNA*_c.1488_1488+9del. This variant was inherited from her mother who showed a minor muscular impairment. No history of any muscular disease was documented in previous generations. Patient 11 also carried an additional deletion in *NEB*, a gene associated with myopathies following an autosomal recessive pattern of inheritance. Both their mother and brother carried this *NEB* variant in heterozygosis form and, as expected, showing no symptom to date. In our cohort, three more *de novo* deletions in *LMNA* were identified in patients 4, 9, and 25, where two were diagnosed with L-CMD (patients 4 and 25) and showed slow muscular weakness progression with no cardiac affectation, while patient 9 was diagnosed with EDMD and DCM. In concordance, both deleterious variants were previously reported in L-CMD patients showing phenotypes similar to our patients (Fan et al., 2021).

Patient 18, diagnosed with EDMD, showed slow muscular weakness progression and no cardiac affectation. The patient carried *LMNA*_p.Gln36His, inherited from the father who showed a minor muscular impairment. No history of any muscular disease was documented in previous generations. This variant was never reported, to the best of our knowledge. This patient also carried the deleterious variant *GAA*_p.Asp645Asn, previously identified and associated with GSD (CM980804) (McCready et al., 2007). This *GAA* variant was inherited during heterozygosis from the healthy mother, not showing any symptom of GSD or Pompe disease. In addition, this patient also carried a deleterious indel in *COL6A3.* This variant has not been reported so far, and the gene is associated with dystonia and muscular dystrophy. The variant was inherited from the father who showed a minor muscular impairment. As mentioned above, no history of any muscular disease was documented in previous generations.

Patient 22, diagnosed with EDMD, showed slow muscular weakness progression and no cardiac affectation. The *LMNA*_p.Glu31Lys variant was *de novo.* Despite no aggressive phenotype, the patient died at 10 years old due to severe respiratory infection. This variant has been recently reported in one patient diagnosed with L-CMD, slow muscular weakness progression, and no cardiac affectation (Fan et al., 2021). This patient also carried a deleterious variant in *DYSF* (p.Arg1849Lys), the gene associated with an autosomal recessive muscular dystrophy. This variant was inherited from the asymptomatic mother, as also observed in the brother who also carried the same *DYSF* variant. No history of any muscular disease was documented in previous generations. Finally, patients 13 and 16, both diagnosed with EDMD, DCM and with an ICD, carried only one deleterious variant in *LMNA* (p.Leu38Phe and p.Arg455Pro, respectively). Both rare variants are novel and *de novo*, after segregation of both variants in relatives.

In conclusion, laminopathies associated with muscular disorders are a group of heterogeneous conditions with different onset and development. We suggest that a targeted genetic diagnosis including *LMNA* as well as other genes related to muscular diseases may help to unravel additional potential rare variants that could be associated with more severe phenotypes. However, translation into clinical practice should be performed with caution due to further studies in large cohorts are necessary to clarify role additional variants.

## 5 Limitations

The study had a few limitations. First was the reduced cohort. Due to the rarity of the disease worldwide, it is difficult get enough number of families to obtained a conclusive result in a genotype-phenotype correlation. Therefore, despite reduced number of patients, our cohort of 26 patients is the largest reported so far, other than the 84 patients reported by Fan et al. (2021). Other limitation is the potential pathophysiological role of additional genetic alterations located in other genes not included in our NGS custom-panel and that could be implicated in phenotype modification. A potential future approach is to perform whole exome sequencing and/or whole genome sequencing to identify new alteration in any region of the genome. Our study includes a comprehensive genotype-phenotype correlation in relatives, at our point of view the main fact in genetic interpretation and clinical translation of genetic variants identified. However, both *in vivo* and *in vitro* studies should be also performed to clarify the pathophysiological mechanism associated with the progressive disable phenotype associated with the disease. Therefore, classification of rare variants should be done following ACMG recommendations and should be periodically reanalyzed, particularly if classified as having an ambiguous role. A periodic update of previous classification may help to clarify role of rare variants, helping to clinicians to obtain genetic diagnosis and, if appropriate, adopt preventive measures.

## Data Availability

The raw data supporting the conclusion of this article will be made available by the authors, without undue reservation.
